# The role of trophic, mutualistic, and competitive interactions in an industrial symbiosis process implementation: an ecological network perspective

**DOI:** 10.1007/s11356-024-33454-z

**Published:** 2024-06-08

**Authors:** Diana Escandon-Barbosa, Jairo Salas-Paramo, Victor Castrillon Paque

**Affiliations:** Faculty of Economic Sciences and Administration, Pontificia Universidad Javeriana-Cali, Santiago de Cali, Colombia

**Keywords:** Collaboration networks, Trophic interactions, Mutualistic interactions, Industrial symbiosis, Competitive interactions

## Abstract

For both government and private institutions, the development of collaboration networks becomes an element of great importance for the implementation of related policies such as the circular economy and sustainable practices in manufacturing. Despite the above, such initiatives have not received as much attention in literature but have been decisive as both public and private initiatives. Initiatives in Latin America do not escape this scenario, especially in the creation of conditions that allow the promotion of approaches such as industrial symbiosis. In this way, the present research is aimed at identifying the role of trophic, mutualistic, and competitive interactions in an industrial symbiosis process implementation. A network analysis model is used to achieve this purpose. This technique allows us to know the degree of importance of the different actors that are part of a network, as well as the factors that determine the implementation of initiatives such as industrial symbiosis. Among the results are that empirical findings confirm the presence of trophic interactions that enhance resource efficiency, mutualistic interactions fostering collaboration and synergy, and competitive interactions promoting efficiency and dynamism. Additionally, a green culture, business size, and innovation activities are revealed as influential factors amplifying network dynamics.

## Introduction

For both Europe and countries in America, the development of business models that consider industrial and environmental sustainability has attracted attention in an incremental way (Akrivou et al. 2021). Proof of the above is the need to find balanced forms of demands for water, inputs, and the accumulation of waste that has been the object of study by industrial symbiosis. In this way, industrial symbiosis comes together in a way from which firms can evaluate opportunities to develop efficient processes, create collaborative networks for the reuse of waste, and constantly search for new ways of producing cleanly.

According to Shan et al. (2023), industrial symbiosis is considered an effective way to achieve efficiency in resource use. Likewise, it allows alleviating the problem of carbon emissions and the possibility of implementing regional development to transform economic models into circular models. An important element is that industries that are opposed to this type of actions tend to maintain practices that produce large amounts of pollution and consume large amounts of energy (Dong et al. 2022b, a).

An important aspect to highlight is that companies that are part of an industrial symbiosis network must not only consider risk factors external to them but also to the set of relationships between the companies of which they are part (Massari and Giannoccaro 2022). In this way, the state of the nodes in the network of relationships plays a fundamental role in the way the stability of the network is achieved (Shan et al. 2023). In industrial symbiosis, networks help firms adequately deal with risks, external factors, and deficiencies in operations that could be limitations in the provision of inputs that may affect production (Dong et al. 2022b, a).

From the field of knowledge of industrial symbiosis, some lines of work currently receiving great attention can be evident, such as waste management (Yadav and Majumdar 2023; Moser and Jauschnik 2023; Shi et al. 2023; Noori et al. 2023), network (Yazıcı et al. 2023; Shmeleva et al. 2023; Yu et al. 2023; Ratsimandresy and Miemczyk 2023; Mallawaarachchi et al. 2023; Yang et al. 2023; Wang, et al. 2023; Shan et al. 2023), management (Agudo et al. 2024; Erol et al. 2023; Harfeldt-Berg and Harfeldt-Berg 2023; Bruel and Godina 2023; Hariyani and Mishra 2023; Xue et al. 2023), technology adoption (Beaurain et al. 2023), and circular economy (Bui et al. 2023; Tsolakis et al. 2023; Faria et al. 2023; Liu et al. 2023).

Considering the previous literature, especially in relation to the networks that support industrial symbiosis, two main gaps can be extracted. The first is related to the need to investigate the configuration and structures of the networks related to industrial symbiosis initiatives in terms of size that allow generalizing results according to the available information (Asghari et al. 2023). The second gap is related to the barriers or limitations to industrial symbiosis. Although some research has found few factors that limit it, it is important to develop a more detailed evaluation of the barriers according to specific contexts (Harfeldt-Berg and Harfeldt-Berg 2023). According to the above, the purpose of this research is the identification of the moderating effects of symbiosis network strength, innovation, and green culture among the different types of risks associated with the implementation of industrial symbiosis.

To achieve the above purpose, this research is structured as follows. In the first part, a brief summary of the different elements that impact the implementation of industrial symbiosis is made, highlighting the most relevant literature in the field of knowledge. In the second part, a theoretical framework is presented that explains industrial symbiosis and the need for networks as a support platform. In the third part, the methodology is presented, highlighting the analysis method used. The fourth part presents the main results achieved. Finally, the conclusions and main contributions of the study are presented.

## Theoretical framework

### Ecological network theory

From the perspective of ecological network theory, it is possible to integrate the possibility of understanding human behavior, industrial symbiosis, and the search for environmental sustainability. Different studies in the field of ecological network theory integrate disciplines such as psychology, the biological perspective, and sustainability. From the psychological point of view, it provides a look at how individuals and organizations can make decisions that allow them to adapt to change. This element is a fundamental platform on which industrial symbiosis is based. From the point of view of biology, the ecological network theory allows us to consider the interconnection of ecosystems, highlighting the broad context in which industrial processes can be developed. And from sustainability, industrial symbiosis emerges as a conceptual framework in which collaborative work is prioritized, sustainable practices that allow the search for benefits for the environment while creating a context in which the psychological factors that determine the behavior and decision making that contributes to a sustainable industrial environment.

One of the perspectives that best allows us to describe the interaction between different communities and their structure is ecological network theory. This perspective has been the object of attention because it has elements of analysis that are necessary to understand the way in which populations interact with other population networks (Brose 2010).

In principle, the emphasis of ecological network theory was based on understanding the rules that govern the structure of the network, trying to predict behaviors that relate to each component of the structure (Williams and Martinez 2008). Other more recent approaches to this perspective have discovered certain nodes in the network that are decisive for its maintenance (Petchey et al. 2008).

For scholars such as Landi et al. (2018), the ecological network describes different types of interaction between the species that belong to a community. These interactions can occur of different types such as trophic interactions (feeding), mutualistic interactions (pollination, seed dispersal), and competitive interactions (interference over common resources). In this way, the ecological network theory is composed of a set of nodes that characterize the interactions between species.

For the specific case of trophic networks, the interactions of species are characterized by providing resources to others. Therefore, the other species benefit through the intensity of the interaction. In the case of mutualistic interactions, the species seek to mutually benefit from the interaction developed.

Studies in the field have focused their efforts on facing theoretical challenges related to the complexity and stability of systems. These challenges are based on the need to know less about the way in which interactions in networks assume different architectures and types of communication between species (Lurgi et al. 2016). A theoretical framework that allows these characteristics to be incorporated makes it easier to compare the mechanisms that exist and the behavioral patterns of the species.

Other research in the field of ecology network theory has found that both trophic vs mutualistic relationships can have a degree of influence on the stability of the communities that are part of the network (Mougi and Kondoh 2012). On the other hand, for scholars such as Sauve et al. (2014), there are other types of interactions that within the same network increase the importance of other indirect effects that may be important in the interactions between nodes.

Many of the organizational behavior patterns observed organizational behavior patterns that are observed show complex fluctuations in population dynamics and species relationships (Solé et al. 2002). This type of phenomena allows us to deepen the understanding and knowledge of the populations. The theoretical models that emerge begin to consider spaces and dynamics that restrict movement and interaction between species.

In this way, the present research attempts to contribute to understanding the way in which trophic interactions are really important to ensure interactions between the components of the network, especially when it comes to industrial symbiosis. Likewise, mutualistic interactions allow not only to include the different actors of the component but also to commit them to the dynamics that support the sustainable objectives of the network. Finally, each network will require competitive interaction processes that improve performance in terms of efficiency that will impact all actors and components of the network.

### Industrial symbiosis and ecological network theory

According to Dong et al. (2022b, a), the performance of industrial symbiosis can be impacted by ecological network theory, through the integration of benefits into economic, environmental, and social results. Despite the above, in literature, it is possible to find that studies of industrial symbiosis have been separated from the notion of network.

An important aspect to note is that there is a large number of tools and indicators that account for the sustainability and impact of the network of different types of institutions, including government institutions and different industry stakeholders. In this way, an attempt is made to monitor the performance of industrial parks, in terms of their efficiency in the use of resources as well as their resilience in the implementation of industrial symbiosis (Fan and Fang 2020).

According to scholars such as Dong et al. (2017) and Bian et al. (2020), eco-industrial parks become clusters that are suitable terrain for the management of industrial symbiosis. This process in itself leads to the improvement of the efficient use of resources, while allowing the closure of material loops in both recycling and energy recovery. At the same time, these types of processes will allow reducing costs in the exchange of resources through sharing an infrastructure that stimulates levels of collaboration at the sectoral level (Bian et al. 2020).

On the other hand, industrial symbiosis focuses systematically on supporting an industrial sustainability system between two or more companies collaborating with a certain geographical proximity. In this process, it largely promotes the promotion of true technologies and their transfer for use in the market (Kim et al. 2018). According to Dong et al. (2022b, a), ecological network theory and industrial symbiosis are intertwined in several points, namely, (a) the expansion of the industrial symbiosis network, which indicates the type of synergies that exist in the network. (b) The change in the dynamics of input and energy use related to the environmental and economic impact.

In the implementation of industrial symbiosis, the possibility of roles between different actors comes into play that supports the formation of a dynamic network within an industrial cluster. In this way, industrial symbiosis becomes an instrument that influences the flow between different nodes of the network, thus improving synergy. This is how synergies allow us to consider the context in which the consumption of resources and the generation of waste have a great impact from the implementation of instruments such as industrial symbiosis.

This is how the ecological network allows us to understand not only the different roles assumed in the dynamics of the network (Brose 2010). These roles are aligned with the different types of connections such as the trophic in charge of supplying resources to the network, which allow an improvement in the efficiency of their use and a reduction in the amount of waste. Mutualistic emphasizes the way in which the different actors in the network manage to establish a set of dynamic relationships that allow them to increase synergy and the inclusion of new members in the network (Landi et al. 2018). Finally, the competitive one is responsible for increasing the commitment to the results of the network, while its interconnection allows not only individual improvement but also the improvement of the collective of relationships that are part of the network (Xue et al. 2023). According to the above, the following hypotheses are proposed:

- H1. There is a trophic interaction in industrial symbiosis processes.
- H2. There is a mutualistic interaction in the processes of industrial symbiosis.
- H3. There is a competitive interaction in the processes of industrial symbiosis.

## Methodology

### Data

Between August and September 2022, 400 businesses in six locations around Colombia participated in this research. According to DANE (2022), 67% of business activity in Colombia are concentrated in the following cities: Bogota, Medellin, Cali, Barranquilla, Cartagena, and Santander. Managers from firms in a number of productive sectors participated in the survey. A polling firm was hired to carry out the survey, and in order to assure its accuracy, the corporation was approached to inquire about its interest in taking part. Additionally, in-person interviews were also done.

The examined businesses revealed that 70% of them use natural resources heavily, including electricity, water, gas, and coal. 25% of them said that their garbage is utilized as raw materials in other sectors, and 65% of them indicated that their waste is employed in new production processes inside their own businesses. According to the report, industrial symbiosis was accomplished in 80% of cases because the firm owners thought using sustainable techniques had advantages.

### Variables

In general terms, companies that have engaged in industrial symbiosis are compared with those that have not to understand the composition of networks and their types of interaction. To test the hypotheses posed in this research, variables related to company characterization are considered: company size, sector, and participation in innovation activities and green culture. Additionally, entrepreneurs are asked about the types of networks they have utilized during their industrial symbiosis process: governmental, sectoral, institutions supporting innovation, institutions providing financial support, entrepreneurship support institutions, and civil society (Escandon-Barbosa et al. 2021).

Examining organizational transformation goals for waste reduction, green culture assesses environmental responsibility. A dichotomous variable that probes the initial phase of innovation—when a business commits financial resources to creating novel goods or ground-breaking organizational procedures—measures the degree to which an organization is innovation-oriented. As a consequence, there are two levels of innovation orientation: strong if the firm considers spending resources in the development of innovative activities and weak if the company thinks it is investing in insufficient resources.

For the determination of the existence of trophic interactions, it is established by those participants who supply resources to sustain the network in the long term. Therefore, they tend to create a greater number of relationships around them with high significance.

On the other hand, mutualistic interactions in the processes of industrial symbiosis are those actors that stimulate the creation of additional nodes or relationships to sustain the network. Therefore, they are visualized through a large number of relationships revolving around them, although not necessarily with a high level of significance as generated in trophic interactions. Finally, competitive interactions in the processes of industrial symbiosis enable the overall network’s efficiency to increase and drive the others. Therefore, in the network, they are visualized as negative relationships among different actors.

### Model

To obtain an understanding of the characteristics and behavior of the industrial symbiosis network in Colombia, two points of view were used in the analysis. The first point of view involved taking the network as a whole, with the nodes representing the various stakeholders who participate in the industrial symbiosis network. For the purposes of this study, the directors of each company were asked which other national stakeholders they maintained relationships with as part of the symbiosis’s activities. By using this information which compares several businesses collectively, some indicators were estimated. They are trophic, mutualistic, and competitive interactions.

## Results

The four hundred companies spread out around the country are represented by 14 nodes or actors that make up the industrial network symbiosis Colombia. The next diagrammatic representation of the network shows the connections between nodes, the various unidirectional and bidirectional flows, and any nodes that have no actor references.

**Fig. 1:**
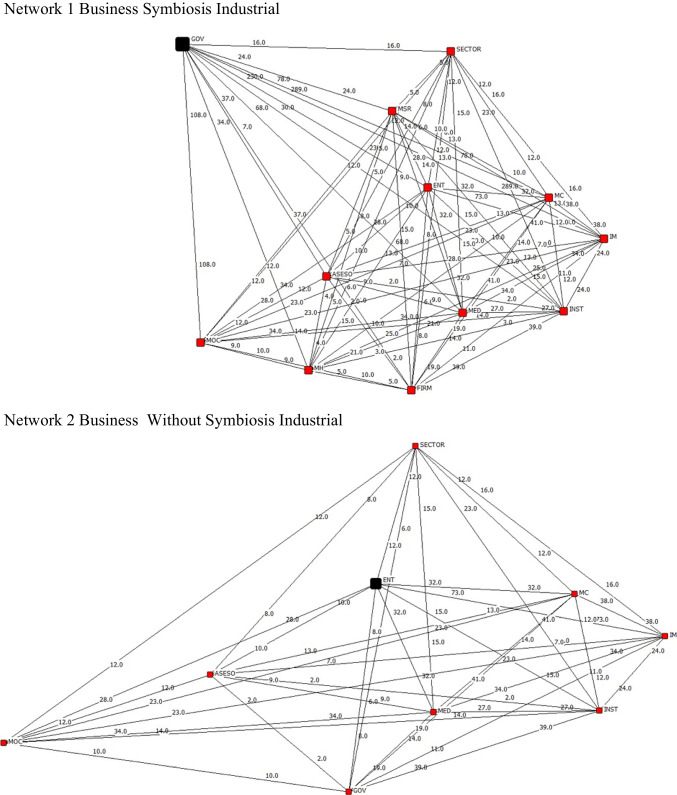
Network 1: Business symbiosis industrial. Network 2: Business without symbiosis industrial

The relationships between the various companies are shown in Fig. 1 in order to create industrial symbiosis. These relationships include institutions that provide business support, social enterprises that allow for symbiosis, and government entities that have an impact on the company’s business activities.

The results of our analysis reveal significant differences in the network strength and composition between businesses that actively engage in industrial symbiosis and those that do not. Industrial symbiosis appears to foster stronger network relationships and a distinct actor composition. Moreover, the influence of green culture, business size, sector, and innovation activities on network characteristics is noteworthy.

In businesses that practice industrial symbiosis, the network exhibits higher levels of connectivity and interdependence among its members, indicating a stronger and more resilient network compared to businesses without industrial symbiosis (*p* < 0.05). This heightened network strength is particularly associated with the prevalence of a green culture (*p* < 0.05), where sustainability practices and environmental consciousness play a central role in shaping the network dynamics.

In terms of business size, larger businesses within the network tend to exert a more pronounced influence (*p* < 0.05). These larger businesses often act as key nodes, facilitating resource exchange and cooperation within the network. Additionally, innovation activities (*p* < 0.05) are more common in businesses with industrial symbiosis, leading to a more dynamic and forward-thinking network environment.

### Actor composition

In businesses with industrial symbiosis, the main actor influencing the network dynamics is the government. The government plays a central role in facilitating and regulating the exchange of resources and fostering cooperation among various businesses. This government involvement contributes to the overall sustainability and resilience of the network. Additionally, green culture is a fundamental aspect within these businesses, with a strong emphasis on environmentally responsible practices that align with the network’s sustainability objectives.

Conversely, in businesses without industrial symbiosis, the primary actor shaping network dynamics is the business itself. These businesses operate more independently, with fewer external connections. This self-reliance may limit their potential for network expansion and the realization of sustainability objectives. Therefore, green culture is less pronounced in businesses without industrial symbiosis, as they tend to focus more on traditional business practices, often with less emphasis on environmental sustainability.

### Types of interactions

Our analysis also indicates that the presence or absence of industrial symbiosis has a discernible impact on the types of interactions within the network, with trophic, mutualistic, and competitive interactions being more pronounced in businesses with industrial symbiosis. These interactions are influenced by business size, sector, and innovation activities.

### Trophic interactions

In businesses with industrial symbiosis, trophic interactions, characterized by the supply of resources, are prevalent. The exchange of resources is highly efficient, leading to a reduction in resource waste and an increase in resource utilization (*p* < 0.05).

Business size has a direct effect on the trophic interactions, with larger businesses playing a pivotal role in resource supply and utilization. Furthermore, innovation activities (*p* < 0.05) within these businesses contribute to the efficient use of resources and a reduction in waste.

### Mutualistic interactions

The presence of industrial symbiosis encourages mutualistic interactions, where different actors within the network establish dynamic relationships to foster collaboration and increase synergy (*p* < 0.05). Sector-specific factors play a role in mutualistic interactions, with businesses in certain sectors exhibiting a greater tendency to engage in mutualistic relationships. Additionally, innovation activities contribute to the development of mutually beneficial connections within the network.

### Competitive interactions

In the context of industrial symbiosis, competitive interactions focus on enhancing the commitment of network members to achieving common goals. These interactions not only lead to individual improvements but also contribute to the collective advancement of the network. Additionally, size of businesses and innovation activities influence competitive interactions, as larger businesses and those engaged in innovation tend to actively participate in competitive processes within the network. Finally, in businesses without industrial symbiosis, the observed network interactions are generally less complex, with trophic interactions being the dominant pattern. The absence of strong mutualistic and competitive interactions may limit the overall sustainability and efficiency of the network.

## Conclusions

This study embarked on a journey informed by the rich tapestry of ecological network theory, aiming to unravel the interplay of human behavior, industrial symbiosis, and sustainability. The theoretical foundation laid the groundwork for an in-depth exploration of these dimensions, with a particular focus on the hypotheses proposed. The amalgamation of these theoretical insights with the empirical results has unveiled a compelling narrative that advances our understanding of sustainable networks and their intricacies.

Hypothesis H1 posited the presence of trophic interactions in industrial symbiosis processes. The empirical findings affirm this hypothesis, demonstrating that businesses engaged in industrial symbiosis indeed exhibit a web of trophic interactions, characterized by the efficient supply of resources. This echoes the principles of ecological network theory, where resource exchange is fundamental to network stability and sustainability (Brose 2010; Lurgi et al. 2016). These interactions, rooted in the concept of trophic networks, reduce resource waste, enhance resource utilization, and contribute to the overall efficiency of the network (Mougi and Kondoh 2012).

The empirical results unequivocally confirm the hypothesis that the presence of mutualistic interactions in the processes of industrial symbiosis. Industrial symbiosis fosters dynamic relationships among network actors, leading to mutualistic interactions that drive collaboration and synergy (Landi et al. 2018). The concept of mutualistic interactions parallels the intricate web of species interactions within ecological networks, emphasizing the interconnectedness and inclusivity of network members (Mougi and Kondoh 2012). These mutualistic interactions play a pivotal role in expanding the network and facilitating collective advancements.

Finally, the empirical evidence supports the hypothesis about the presence of competitive interactions in the processes of industrial symbiosis. Competitive interactions, driven by the commitment of network members to common objectives, manifest in businesses actively engaged in industrial symbiosis. These interactions, reminiscent of the competitive processes within ecological networks, lead to both individual and collective improvements (Xue et al. 2023). The heightened efficiency and dynamism resulting from competitive interactions benefit the sustainability and resilience of the industrial symbiosis network.

In light of these findings, the theoretical framework and empirical results converge to emphasize the fundamental role of industrial symbiosis in the development of strong, sustainable networks. The presence of a green culture, characterized by a deep commitment to sustainability and environmental responsibility, underpins the strength of networks within businesses engaged in industrial symbiosis. Moreover, the size of businesses and their dedication to innovation activities further amplify network dynamics.

These findings not only underscore the theoretical underpinnings of ecological network theory but also provide empirical validation of the theoretical constructs. As the world’s concerns for environmental conservation intensify, this research champions the adoption of industrial symbiosis principles and the cultivation of a green culture as essential strategies for fostering environmentally sustainable networks. The multifaceted relationships between ecological network theory, industrial symbiosis, and sustainability offer profound insights and provide a foundation for businesses, policymakers, and researchers committed to advancing the dual causes of environmental preservation and economic prosperity (Brose 2010; Lurgi et al. 2016; Landi et al. 2018; Xue et al. 2023).

### Management contributions

In the realm of management, the findings from this study offer significant contributions. They underscore the pivotal role of strategic decision-making in the adoption of industrial symbiosis practices, prompting management teams to incorporate sustainability into their strategic planning. By fostering a green culture within their organizations, management can not only advance sustainability objectives but also bolster the strength and resilience of networked collaborations. Additionally, these insights underline the potential for industrial symbiosis to enhance sustainability, encouraging management to prioritize and justify sustainability initiatives while capitalizing on the advantages of resource efficiency and waste reduction that these networks offer.

The research also emphasizes the importance of resource optimization, as efficient resource exchange within industrial symbiosis networks can lead to cost savings and improved environmental performance. Furthermore, management is prompted to harness the power of innovation and collaboration to drive efficiency, sustainability, and competitiveness. Understanding the significance of stakeholder engagement within network dynamics, management can prioritize building and sustaining strong relationships with stakeholders, including government agencies. Lastly, by recognizing the limitations and potential external factors that may impact network dynamics, management can develop robust risk management strategies to adapt to changing conditions and safeguard their organization’s sustainability efforts. These insights collectively empower management to make informed decisions that foster environmentally responsible, efficient, and resilient businesses in an ever-evolving global landscape.

### Future lines

Future research in this field can follow several promising paths. Longitudinal studies can investigate the enduring impact of industrial symbiosis on network dynamics and sustainability, providing insights into network evolution over time. Cross-sector analyses can reveal sector-specific patterns within industrial symbiosis networks, offering a comprehensive understanding of their applicability in diverse contexts. Global comparisons across regions and countries may help identify the influence of cultural, regulatory, and economic factors on industrial symbiosis adoption, providing a global perspective on network sustainability. Further research can delve into environmental impact assessments, quantifying benefits like reduced emissions and waste, enhancing our understanding of the ecological advantages of networked sustainability initiatives. Additionally, studying stakeholder engagement within these networks can shed light on how different actors contribute to sustainability objectives.

### Limitations

While this study offers valuable insights, it has inherent limitations. Generalizability is limited, as the study focused on a specific context, and its findings may not directly apply to other regions or sectors. Data availability and quality may also affect the depth of analysis. Causation-versus-correlation complexities remain, necessitating further research to establish causal relationships among network characteristics and industrial symbiosis. Subjectivity and bias may influence interpretations of network dynamics, green culture, and the roles of business size, sector, and innovation activities. The study offers a snapshot, not accounting for network evolution. Complex interactions within ecological network theory may be oversimplified, and unexamined external factors, such as economic and regulatory changes, may affect network dynamics. Acknowledging and addressing these limitations will pave the way for a more comprehensive understanding of ecological network theory and industrial symbiosis in advancing sustainable networks and a greener future.

## References

[CR1] Agudo FL, Bezerra BS, Gobbo Júnior JA (2024). An overview of Brazilian companies' readiness to implement industrial symbiosis. Bus Strateg Environ.

[CR2] Akrivou C, Łȩkawska-Andrinopoulou L, Tsimiklis G, Amditis A (2021). Industrial symbiosis platforms for synergy identification and their most important data points: a systematic review. Open Res Europe.

[CR3] Asghari M, Afshari H, Jaber MY, Searcy C (2023). Dynamic deployment of energy symbiosis networks integrated with organic Rankine cycle systems. Renew Sustain Energy Rev.

[CR4] Beaurain C, Chembessi C, Rajaonson J (2023). Investigating the cultural dimension of circular economy: a pragmatist perspective. J Clean Prod.

[CR5] Bian Y, Dong L, Liu Z, Zhang L (2020). A sectoral eco-efficiency analysis on urban-industrial symbiosis. Sustainability.

[CR6] Brose U (2010). Improving nature conservancy strategies by ecological network theory. Basic Appl Ecol.

[CR7] Bruel A, Godina R (2023). A smart contract architecture framework for successful industrial symbiosis applications using blockchain technology. Sustainability.

[CR8] Bui TD, Tseng JW, Tran TPT, Ha HM, Lim MK, Tseng ML (2023) Circular supply chain strategy in Industry 4.0: the canned food industry in Vietnam. Business Strategy Environ

[CR9] Dong L, Liang H, Zhang L, Liu Z, Gao Z, Hu M (2017). Highlighting regional eco-industrial development: life cycle benefits of an urban industrial symbiosis and implications in China. Ecol Model.

[CR10] Dong L, Taka GN, Lee D, Park Y, Park HS (2022). Tracking industrial symbiosis performance with ecological network approach integrating economic and environmental benefits analysis. Resour Conserv Recy.

[CR11] Dong L, Taka GN, Lee D, Park Y, Park HS (2022). Tracking industrial symbiosis performance with ecological network approach integrating economic and environmental benefits analysis. Resour Conserv Recycl.

[CR12] Erol I, Peker I, Ar IM, Searcy C (2023). Examining the role of urban-industrial symbiosis in the circular economy: an approach based on N-Force field theory of change and N-ISM-Micmac. Oper Manag Res.

[CR13] Escandon-Barbosa D, Salas-Paramo J, Meneses-Franco AI, Giraldo-Gonzalez C (2021). Adoption of new technologies in developing countries: the autonomous car between Vietnam and Colombia. Technol Soc.

[CR14] Fan Y, Fang C (2020). Assessing environmental performance of eco-industrial development in industrial parks. Waste Manage.

[CR15] Faria E, Barreto C, Caldeira-Pires A, Streit JAC, Guarnieri P (2023). Brazilian circular economy pilot project: integrating local stakeholders’ perception and social context in industrial symbiosis analyses. Sustainability.

[CR16] Harfeldt-Berg L, Harfeldt-Berg M (2023). Connecting organizational context to environmental sustainability initiatives and industrial symbiosis: empirical results and case analysis. Sustain Prod Consum.

[CR17] Hariyani D, Mishra S (2023) A descriptive statistical analysis of enablers for integrated sustainable-green-lean-six sigma-agile manufacturing system (ISGLSAMS) in Indian manufacturing industries. Benchmarking: An International Journal, (ahead-of-print)

[CR18] Kim HW, Dong L, Jung S, Park HS (2018). The Role of the Eco-Industrial Park (EIP) at the national economy: an input-output analysis on Korea. Sustainability.

[CR19] Landi P, Minoarivelo HO, Brännström Å, Hui C, Dieckmann U (2018). Complexity and stability of ecological networks: a review of the theory. Popul Ecol.

[CR20] Liu Z, Hansen DW, Chen Z (2023). Leveraging digital twins to support industrial symbiosis networks: a case study in the Norwegian wood supply chain collaboration. Sustainability.

[CR21] Lurgi M, Montoya D, Montoya JM (2016). The effects of space and diversity of interaction types on the stability of complex ecological networks. Thyroid Res.

[CR22] Mallawaarachchi H, Karunasena G, Sandanayake Y, Liu C (2023). Conceptualising a model to assess the optimum water flow of industrial symbiosis (IS). Sustainability.

[CR23] Massari GF, Giannoccaro I (2022). Simulating the network structures in the circular economy and their impact on resilience. IFAC-PapersOnLine.

[CR24] Moser S, Jauschnik G (2023). Using industrial waste heat in district heating: insights on effective project initiation and business models. Sustainability.

[CR25] Mougi A, Kondoh M (2012). Diversity of interaction types and ecological community stability. Science.

[CR26] Noori S, Korevaar G, Stikkelman R, Ramírez A (2023). Exploring the emergence of waste recovery and exchange in industrial clusters. J Ind Ecol.

[CR27] Petchey OL, Beckerman AP, Riede JO, Warren PH (2008). Size, foraging, and food web structure. Proc Natl Acad Sci.

[CR28] Ratsimandresy A, Miemczyk J (2023). Conceptualising collaborations beyond industrial boundaries: a literature review and a theoretical proposition to understand cross-industrial collaborations in the circular supply network. Sustainability.

[CR29] Sauve AM, Fontaine C, Thébault E (2014). Structure–stability relationships in networks combining mutualistic and antagonistic interactions. Oikos.

[CR30] Shan H, Guo Q, Wei J (2023). The impact of disclosure of risk information on risk propagation in the industrial symbiosis network. Environ Sci Pollut Res.

[CR31] Shi Y, Lin Y, Wang S, Wen H, Lim MK, Tseng ML (2023). Resource saving and carbon footprint reduction potential of urban symbiosis strategy in express packaging waste recycling network. Waste Manage.

[CR32] Shmeleva N, Tolstykh T, Dudareva O (2023). Integration as a driver of enterprise sustainability: the Russian experience. Sustainability.

[CR33] Solé RV, Alonso D, McKane A (2002). Self–organized instability in complex ecosystems. Philos Trans R Soc Lond Series B: Biol Sci.

[CR34] Tsolakis N, Goldsmith AT, Aivazidou E, Kumar M (2023). Microalgae-based circular supply chain configurations using Industry 4.0 technologies for pharmaceuticals. J Clean Prod.

[CR35] Wang L, Zhang Q, Zhang G, Wang D, Liu C (2023). Can industrial symbiosis policies be effective? Evidence from the nationwide industrial symbiosis system in China. J Environ Manage.

[CR36] Williams RJ, Martinez ND (2008). Success and its limits among structural models of complex food webs. J Anim Ecol.

[CR37] Xue X, Wang S, Chun T, Xin H, Xue R, Tian X, Zhang R (2023). An integrated framework for industrial symbiosis performance evaluation in an energy-intensive industrial park in China. Environ Sci Pollut Res.

[CR38] Yadav VS, Majumdar A (2023). Mitigating the barriers of industrial symbiosis for waste management: an integrated decisionmaking framework for the textile and clothing industry. Waste Manag Res.

[CR39] Yang L, Wang Y, Zhang W, Tan Z, Anwar SU (2023). Optimal pricing and sourcing strategies in a symbiotic supply chain under supply uncertainty. J Clean Prod.

[CR40] Yazıcı E, Alakaş HM, Eren T (2023). Prioritizing of sectors for establishing a sustainable industrial symbiosis network with Pythagorean fuzzy AHP-Pythagorean fuzzy TOPSIS method: a case of industrial park in Ankara. Environ Sci Pollut Res.

[CR41] Yu H, Da L, Li Y, Chen Y, Geng Q, Jia Z, ... & Gao C (2023) Industrial symbiosis promoting material exchanges in Ulan Buh Demonstration Eco-industrial Park: a multi-objective MILP model. J Clean Prod 414: 137578

